# Effect of mild blast-induced TBI on dendritic architecture of the cortex and hippocampus in the mouse

**DOI:** 10.1038/s41598-020-59252-4

**Published:** 2020-02-10

**Authors:** Whitney A. Ratliff, Ronald F. Mervis, Bruce A. Citron, Brian Schwartz, Vardit Rubovitch, Shaul Schreiber, Chaim G. Pick

**Affiliations:** 1 Bay Pines VA Healthcare System, Research and Development, Bay Pines, FL USA; 2 NeuroStructural Analytics, Inc., Columbus, OH USA; 3 Center for Aging and Brain Repair, Department of Neurosurgery and Brain Repair, University of South Florida Morsani College of Medicine, Tampa, FL USA; 4 VA New Jersey Health Care System, Research & Development, East Orange, NJ USA; 5 Department of Pharmacology, Physiology, and Neuroscience, Rutgers- New Jersey Medical School, Newark, NJ USA; 6 Department of Anatomy and Anthropology, Tel Aviv University Sackler School of Medicine, Tel Aviv, Israel; 7 Department of Psychiatry, Tel Aviv Sourasky Medical Center, Tel Aviv, Israel; 8 Department of Psychiatry, Tel Aviv University Sackler School of Medicine, Tel Aviv, Israel; 9 Sagol School of Neuroscience, Tel Aviv University, Tel Aviv, Israel; 10 Dr. Miriam and Sheldon G. Adelson Chair and Center for the Biology of Addictive Diseases, Tel Aviv University, Tel Aviv, Israel

**Keywords:** Cell death in the nervous system, Cellular neuroscience

## Abstract

Traumatic brain injury (TBI) has been designated as a signature injury of modern military conflicts. Blast trauma, in particular, has come to make up a significant portion of the TBIs which are sustained in warzones. Though most TBIs are mild, even mild TBI can induce long term effects, including cognitive and memory deficits. In our study, we utilized a mouse model of mild blast-related TBI (bTBI) to investigate TBI-induced changes within the cortex and hippocampus. We performed rapid Golgi staining on the layer IV and V pyramidal neurons of the parietal cortex and the CA1 basilar tree of the hippocampus and quantified dendritic branching and distribution. We found decreased dendritic branching within both the cortex and hippocampus in injured mice. Within parietal cortex, this decreased branching was most evident within the middle region, while outer and inner regions resembled that of control mice. This study provides important knowledge in the study of how the shockwave associated with a blast explosion impacts different brain regions.

## Introduction

Brain trauma is one of the largest causes of death and disability in the United States, accounting for about 30% of all injury deaths^1^. Traumatic brain injury (TBI) occurs when sufficient external force is imposed on the head resulting in brain damage. TBI varies in severity, with the most common form being mild (mTBI), which accounts for approximately 80% of the cases in the United States^2^. While more severe TBI involve a mix of neuronal cell death and axonal degradation, mild TBIs can produce more subtle morphological changes within the cells of the brain and consequent cognitive deficits for which there are no consistently effective treatments. In addition to this, laboratory and clinical evaluations of mTBI patients (i.e. CT scan, MRI) often fail to reveal any clear and consistent morphological changes to the brain, thus making proper diagnosis difficult^3,4^. Primary mechanical changes due to impact are generally followed by several biologic processes that occur in the minutes and days following mTBI^5^, resulting in secondary brain injuries, including inflammation in the brain, giving rise to elevated intracranial pressure^6^. Physical symptoms of mTBI typically include headaches, nausea, dizziness and weakness; in addition to this there are a number of cognitive symptoms such as amnesia and problems with short term memory, as well as emotional symptoms such as depression or anxiety.

Traumatic brain injury has become an increasingly common affliction among military personnel in recent conflicts. Among U.S. Forces, there have been approximately 384,000 brain injuries sustained since 2000^7^. Blast-related TBIs make up a significant portion of these injuries^8,9^, accounting for numerous long term cognitive deficits in veterans who have been exposed to blasts. In the Iraq and Afghanistan wars, mTBI has been identified as a signature injury with an estimated 10–20% of veterans having been affected^10^. In 2017, over 83,000 military personnel were diagnosed and treated for TBI by the U.S. Department of Defense or the Veterans Administration^11^. These injuries are often the result of exposure to an explosion which generates a blast shockwave that impacts the brain. The resulting injuries have been termed blast-related traumatic brain injuries (bTBI). Data currently suggests that there are major differences which exist in the pathophysiology between blast-related and non-blast related TBI^12–15^. One of the difficulties of assessing blast injuries is the different spectra of clinical manifestations that can appear due to mTBI after a direct blow to the head^16^, blast waves^17^, or a mix of both^18^. One animal study even suggests that mTBI can occur as a result of blast pressure waves directed at the chest of the subject^19^.

Several studies have investigated the effects of mechanical brain trauma on neuronal morphology. Results consistently suggest decreases in neuronal density within the cortex and hippocampus on the ipsilateral side to the injury^20–23^. Interestingly, this dendritic damage extends well beyond the area of injury; relatively minimal cell death still results in significant dendritic changes^24,25^. This may account for the cognitive deficits observed in both humans and animals exposed to a mild TBI. While there is significant evidence to suggest that even mild mechanical TBI induces relatively widespread dendritic changes, there have been few studies investigating these changes as a result of blast TBI.

The purpose of this study was to evaluate and characterize the effects of blast-induced mTBI on the dendritic morphology of neurons in the parietal cortex and hippocampus of an animal model (the mouse brain) at the cellular level. 95% of the volume of the neuron is in the dendritic arbor and changes in dendritic parameters are a very sensitive index of neuronal damage. Neurons were visualized using the Rapid Golgi staining method. This technique randomly stains the soma and the complete dendritic arbor of about 5% of all neurons and was the method of choice to permit visualization and quantification of the dendritic branching. The Golgi Staining technique is a powerful visualization tool in the study of neuroanatomy. Modifications of the original stain continue to be some of the best for small-scale analysis and have become a popular choice of neuroscientists examining the effects of TBI on dendritic morphology over the past six years^26^. Golgi staining, because of its high contrast, completeness in staining and selectivity of less than ten percent of cells can be translated exceptionally with camera lucid drawings as well as light microscopy; these qualities allow images to be compiled from multiple levels of depth without muddiness from neighboring neurons. From these images, numerical characterizations can be made to determine the complexity of dendritic branching including Sholl analysis which counts the amount and length of branches and Dendritic Branching analysis which tracks the amount of branch splitting.

Previous experiments involving our model of blast injury, at the same peak over-pressure, have yielded cognitive and behavioral changes in blast exposed mice similar to that TBI in humans at both acute and chronic timepoints, such as deficits in spatial and recognition memory and increased anxiety-like behavior^27,28^. One previous study also noted significant neurodegeneration within the neurons of the cortex and hippocampus at 3 days post-injury via FluoroJade B staining^29^. The goal of our study was to identify morphological changes within the neurons of the cortex and hippocampus occurring as a result of blast exposure at this timepoint to better understand how small morphological changes occurring within the brain following injury may induce cognitive deficits and how we can identify treatments to prevent these changes from progressing.

## Materials and Methods

### Animal husbandry

Male ICR mice were obtained from Harlan Sprague Dawley Inc. (now Envigo, Jerusalem, Israel) weighing approximately 25–30 g were kept under a constant 12-h light/dark cycle at room temperature, with up to 5 animals per cage. Ad libitum food and water was provided. The ethics committee of the Sackler Faculty of Medicine approved the experimental protocol (M-10-034), in compliance with the guidelines for animal experimentation of the National Institutes of Health. For this experiment, 6 brains from bTBI mice and 4 brains from sham mice were analyzed. Animals from different experimental groups were housed in the same room, but not in the same cages. No instances of fighting prior to or following bTBI exposure was noted.

### Blast trauma model

**Figure 1:**
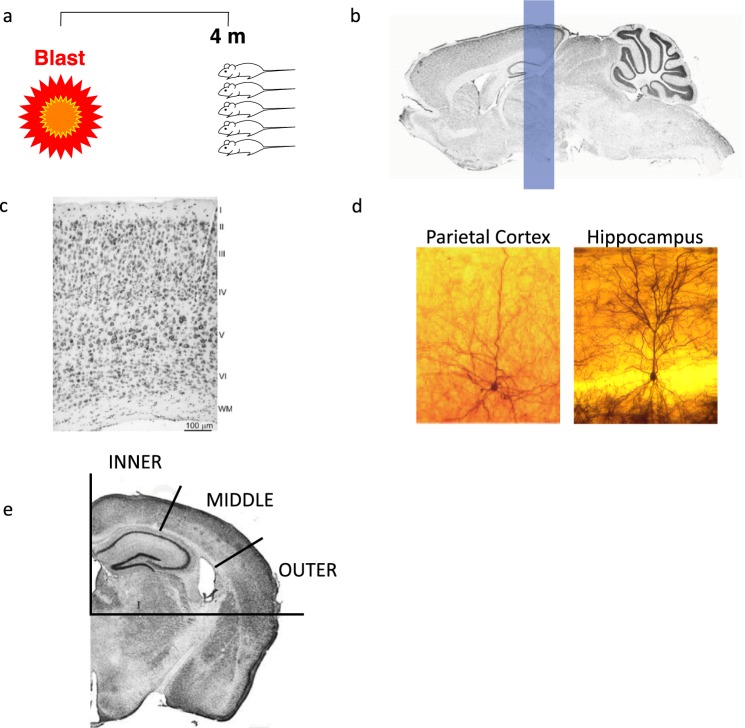
Mouse model of mild blast TBI. (**a**) Prior to blast, anesthetized mice were placed in a net within a holder with adjacent pressure gauges at a distance of 4 m from an explosive charge containing 500 g TNT. The explosion generates approximately 5.5 psi peak over-pressure at this distance. The measured peak over-pressure for this experiment was 37.9 kPa (~5.5 psi). The animals, pressure gauges, and the explosive charge was elevated 1.0 m above ground level. The rows are situated 4 meters from the TNT cast. Each row had space for 12 mice and two pressure gauges were mounted at the ends of each platform. Brain regions were analyzed via Rapid Golgi stain. (**b**) Coronal blocks of tissue incorporating the parietal cortex (and underlying hippocampus) were removed and neurons were Golgi impregnated using the Rapid Golgi method, shown in (**c**) (Mulherkar (2017) (https://www.nature.com/articles/s41598-017-11113-3) under the conditions set forth by Creative Common BY 4.0 license (http://creativecommons.org/licenses/by/4.0/)). Individual Golgi stained neurons of the parietal cortex and hippocampus are shown in (**d**). The parietal cortex was broken down into inner, middle, and outer regions for further analysis (**e**) (Mulherkar (2017) (https://www.nature.com/articles/s41598-017-11113-3) under the conditions set forth by Creative Common BY 4.0 license (http://creativecommons.org/licenses/by/4.0/)).

The blast-induced TBI protocol took place in the experimental site of “Tamar Explosives” and of “Sadwin Consultancy” in central Israel^28^. Mice (both bTBI and sham controls) were anesthetized via I.P. injection with a mixture of ketamine (100 mg/kg) and xylazine (10 mg/kg), a combination that induces deep anesthesia and still enables spontaneous respiration. Mice were placed, with noses facing the source of the explosion device, in a restricted plastic net in a holder which is clamped to the floor with screws. This device maintained their position but allowed exposure to the blast shockwave (Fig. 1a). A gauge measuring blast levels (in PSI) and temperature was secured to each holder next to the mice. The explosion device used was a constructed cast of 500 g TNT. The animals, gauges, and the explosive charge was were elevated 1.0 m above ground level. In order to reduce confounding variables, all animals used in this experiment were exposed to one explosion, during the same trial, such that all experimental animals receive the same blast under the same conditions. Animals were positioned such that the angle and distance relative to the blast were identical. Placed at a distance of 4 m, mice were exposed to 5.5 PSI peak over-pressure during the blast, measured ‘side-on’ with Free-Field ICP® Blast Pressure Sensor “pencil gauges” (PCB Piezoelectronics, Depew, NY, USA; Model 137). Photographs of the blast apparatus and site have been published previously^30^. Following explosion, mice receive an impact of the “direct” shockwave, as well as the reflected wave from the ground. A reflected wave from the embankment surrounding the arena can also be expected, but is likely insignificant relative to the initial direct and reflected waves. Each of these shockwaves combine to form a complex blast injury representative of what would be experienced by a human exposed to an explosive blast. The sampling rate was 61.25 kHz or 16 microseconds between readings on all channels. The pressure-time curves were recorded for each experiment.

### Dendritic analysis

Dendritic analysis was performed as described in a previous study investigating dendritic changes within the amygdala of this mouse cohort following blast^30^. Mice were perfused 72 hours post injury with phosphate buffered saline followed by 4% neutral buffered formalin. Brains were then removed and placed in 10% formalin overnight at 4 °C. The following day, brains were cryoprotected in 15% sucrose and incubated at room temperature for 24 hours. The cortical samples were then incorporated into formalin-fixed tissue blocks (2–3 mm thick in the coronal plane) and stained using the Rapid Golgi method. Fixed tissue blocks were first placed in potassium dichromate and osmium tetroxide for approximately 6 days, then transferred to 0.75% silver nitrate for approximately 40 hours. Increasing concentrations of alcohol solutions and ethyl ether were used to dehydrate the blocks, followed by infiltration with increasing concentrations of nitrocellulose solutions (5%, 10%, 20%, 30%; 1–2 days each). Blocks were then placed in plastic molds and hardened using chloroform vapors. Tissue sections containing the parietal cortex and underlying hippocampus within the region approximately −1.7 mm to −2.7 mm from Bregma (Fig. 1b) were cut to a thickness of 120 microns in the coronal plane using an AO sliding microtome, cleared in alpha-terpineol, rinsed with xylene, and mounted on slides using Permount. Strict selection criteria were used to select neurons for analysis. Neurons must have been well impregnated; branches had to be unobscured by other neurons, glia, blood vessels, or undefined precipitate (a staining by-product), and the soma must have been located within the middle third of the section (Fig. 1c). Representative images of individual Golgi stained neurons for within both the parietal cortex and hippocampus are shown in Fig. 1d. A Zeiss brightfield microscope with long-working distance oil-immersion objective lessee and drawing tubes was used to prepare camera lucida drawings for analysis.

Dendritic arbors were analyzed from both brain hemispheres using two methods: the Sholl Analysis and Branch Point Analysis (BPA), as previously described^31–33^. Sholl analysis defines the mean amount and distribution of the dendritic arbor^34^. Concentric circles or “shells” are superimposed over the camera lucida drawings. The radius of the first shell was 10 μm from the cell soma and subsequent shells increased by 10 μm increments out to 200 μm away from the soma. Interactions are measured by the number of times a dendrite intersects with a shell. BPA measures the complexity of the dendritic arbor by quantifying the number of dendrite branch bifurcations. A first-order branch is a branch emanating directly from the soma. The point where this branch splits into two is the first-order branch point and the resultant branches are second-order branches. This pattern continues until the branches reach an endpoint. More branch points indicate increased dendritic branching complexity.

### Statistical analysis

GraphPad Prism software was used for statistical analysis. Mean values are depicted ± standard deviation and were compared using the two tailed t test. For Sholl and Branch Point Analyses, the Wilcoxon signed-rank test was used with animal n being used for analysis. In both tests *p *< 0.05 indicates significance.

## Results

### Sholl analysis

**Figure 2:**
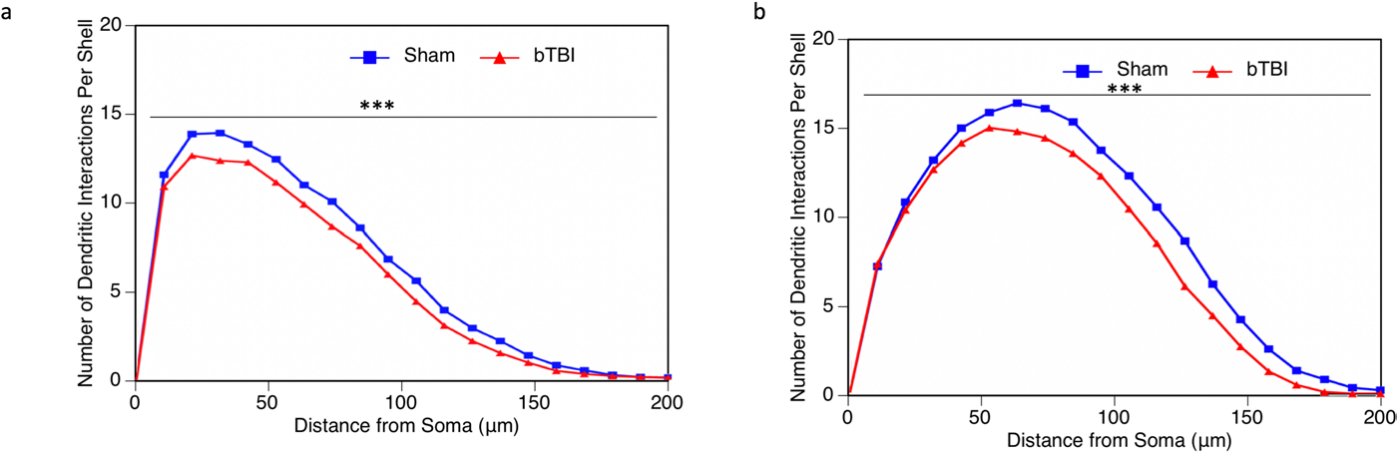
Sholl analysis of parietal layer V and VI pyramidal neurons and hippocampal CA1 neurons. Sholl analysis was performed to evaluate the amount and distribution of the dendritic arbor following bTBI. (**a**) In parietal cortex, there was a significant reduction in the number of dendritic intersections per shell in bTBI mice (n = 6, 54 total neurons analyzed) when compared to sham controls (n = 4, 36 total neurons analyzed) (*p* = 0.0002). (**b**) The same pattern was observed in the hippocampus, with a significant reduction in the number of dendritic intersections per shell in bTBI mice (n = 6, 54 total neurons analyzed) compared to sham controls (n = 4, 36 total neurons analyzed) (*p* < 0.0001).

Sholl Analysis was performed to assess the dendritic distribution of the dendritic arbor at increasing distances from the soma. Within the infragranular layer IV and V pyramidal neurons of the parietal cortex, we saw a highly significant reduction in dendritic intersections per shell as a result of injury (Fig. 2a). The same pattern was observed within the CA1 basilar tree of the hippocampus (Fig. 2b). This suggests that our model of mild bTBI does result in a reduction in the distribution of the dendritic arbor.

### Dendritic length

**Figure 3:**
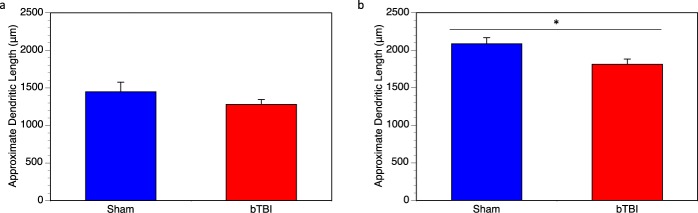
Dendritic length per neuron in cortex and hippocampus. Sholl analysis was also used to approximate the average dendritic length per neuron. (**a**) In cortex, bTBI mice had a slightly reduced average dendritic length per neuron, however, this was not significant (*p* = 0.352). (**b**) In hippocampus, bTBI mice had significantly reduced dendritic length compared to controls (*p* = 0.013).

The data from the Sholl analysis were used to approximate the total dendritic length (in microns). Within the layer IV and V pyramidal neurons, we did not see any significant changes in dendritic length with injury, 1449 ± 127.7 μm (100 ± 8.81%) in the sham mice and 1281 ± 63.83 μm (88.41 ± 4.41%) in the injured mice (Fig. 3a). However, we do see a significant reduction in dendritic length with injury in the hippocampus, 2086 ± 82.04 μm (100 ± 4.12%) in the sham mice and 1813 ± 69.65 μm (86.87 ± 4.55%) in the injured mice (Fig. 3b).

### Branch point analysis

**Figure 4:**
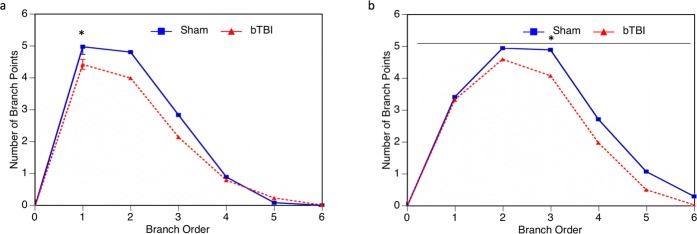
Branch point analysis of cortical and hippocampal neurons. Branch point analysis was used to assess the dendritic complexity of neurons following bTBI based upon the number of dendritic bifurcations at varying branch orders from the soma. (**a**) In parietal cortex, dendritic complexity was reduced in mice who received a bTBI, however, this result was only statistically significant at the first branch order (*p* = 0.0461). (**b**) Dendritic complexity was also significantly reduced in all branch orders within the hippocampus of bTBI mice (*p* = 0.0156).

Branch Point Analysis shows the complexity of the dendritic arbor based on the number of dendritic bifurcations at each branch order. More branch points indicate more dendritic complexity. Branch Point Analysis of neurons within the parietal cortex indicates that there is a reduction in dendritic complexity for the first 4 branch orders in bTBI mice, though this reduction is only statistically significant for the first branch order (Fig. 4a). In hippocampus, we see a significant reduction in branch points for all branch orders (Fig. 4b). Overall, we see that our injury model does induce a reduction in dendritic complexity in both the parietal cortex and hippocampus.

### Analysis of parietal cortex regions

**Figure 5:**
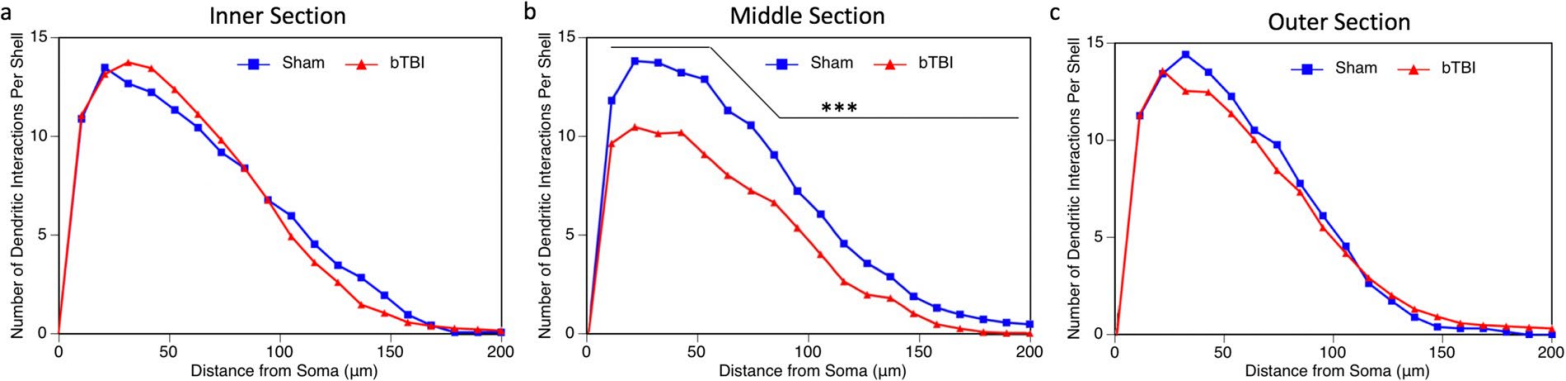
Sholl analysis of parietal regions. Sholl analysis was performed on individual parietal regions, designated as inner, middle, and outer. (**a**) There was no significant change in the dendritic arbor within the inner sector (*p* = 1.00). (**b**) Within the middle sector, there was significantly reduced dendritic interactions in mice receive a bTBI (*p* < 0.0001). (**c**) Similar to the inner sector, there was also no difference between groups in the outer sector (*p* = 0.3443). In all cases, three total neurons were analyzed per mouse, 18 total neurons analyzed for bTBI group and 12 total neurons analyzed for sham controls.

**Figure 6:**
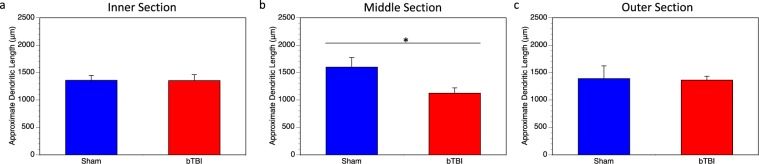
Dendritic length in the inner, middle, and outer sectors of parietal cortex. Sholl analysis was used to approximate the dendritic length within the three regions of the parietal cortex. (**a**) There was no significant difference between bTBI and sham mice in the inner region (*p* = 0.9143). (**b**) The middle region showed a significant reduction in dendritic length in mice receive a bTBI compared to sham controls (*p* = 0.0466). (**c**) There was no significant difference between bTBI and sham mice in the outer region (*p* = 1.00).

To further understand how our model of bTBI is influencing specific areas of the parietal cortex, we divided the parietal cortex into three regions, each representing a third of the parietal cortex: inner, middle, and outer (Fig. 1e). One third of the previously analyzed parietal neurons are represented in each of these regions. When we examine the layer IV and V pyramids from these individual regions using Sholl analysis, we see that there is no loss of the dendritic arbor in the inner (Fig. 5a) and outer (Fig. 5c) regions. There is, however a highly significant reduction in the distribution of the dendritic arbor in the middle region (Fig. 5b), accounting for the reduction shown in the Sholl analysis of the entire region explained previously. Further, we also see a significant reduction in dendritic length per neuron in this middle region, 1599 ± 175.7 μm (100 ± 10.99%) in the sham mice and 1125 ± 95.19 μm (70.36 ± 5.95%) in the injured mice (Fig. 6b). This was not seen in the inner (Fig. 6a) and outer (Fig. 6c) regions. As such, we see that the middle region of the parietal cortex experiences the most damage as a result of the blast injury, despite the entire brain being exposed to the blast.

### Results summary

Overall, we saw that mild bTBI resulted in reduced distribution of the dendritic arbor in both the parietal cortex and in the hippocampus. Further analysis shows that the middle region of the parietal cortex accounts for the majority of this reduction. We also saw that dendritic length was decreased in hippocampus, but not in parietal cortex. However, we do see a decrease in dendritic length when we isolate the middle region of the parietal cortex. Further, we also see a decrease in dendritic complexity in both cortex and hippocampus following mild bTBI.

## Discussion

Our results indicate that mild blast-related TBI induced morphological changes within both the parietal cortex and the hippocampus of the model mice. Sholl and branch point analyses indicated that there was a significant decrease in dendritic distribution and density. This is consistent with previous rodent studies of mechanical TBI^20–23,30^. Our branch point analysis also indicated that there was decreased dendritic branching in parietal cortex and hippocampus. Additionally, we saw region-specific changes within the cortex. The middle section of the parietal cortex appears to make up the majority of the changes in dendritic distribution and density, while the inner and outer sections are relatively unchanged. Unlike controlled mechanical injury, our explosive blast exposes the whole brain to the shockwave. It is interesting that, despite this, damage still appears to be relatively localized to the middle region.

Interactions between the amygdala and hippocampus have been shown to be integral to the association between emotion and memory^35^. In our bTBI mouse model, we see a similar pattern where we have observed increases in dendritic spine density in the basolateral amygdala^30^ coupled with the decreased dendritic branching within the hippocampus observed in this study. Our results within the parietal cortex are also consistent with fMRI studies in humans who have experienced mTBI from both mechanical and blast injury, which indicate decreased activity within that region^36,37^. We saw decreases in dendritic length and complexity which may suggest a decrease in connectivity within the hippocampus. One previous study showed that changes in branch point morphology within the hippocampus had a direct effect on the ability of neurons to generate, propagate, and time action potentials^38^. These observations could also account for the decreases in spatial and recognition memory that we have observed in our mouse model previously^28,29^.

There do seem to be similarities and differences between blast-induced TBI compared to fluid percussion or controlled cortical impacts with respect to the effects on the dendritic arbor. At three days after blast exposure, we observed reduced dendritic arbors, specifically in the pyramidal neurons of layers IV and V of the parietal cortex and in the hippocampal CA1 region. This is in contrast to our previous study in the amygdala, which did not show decreased dendritic arbors or dendritic length as a result of bTBI^30^. Winston *et al*. reported reductions in spine densities in several brain regions in mice at 24 hours after controlled cortical impact and the impact resulted in similar reductions even in the contralateral hemisphere^23^. Rats that received CCI at a young age displayed, at 1 month after injury, the largest reductions (measuring dendritic length and also branch points) in dendritic arbors in the hippocampal CA1 region^22^. Similar results were reported in the thalamus, in rats receiving fluid percussion injury, with a peak reduction in dendritic arbors at 7 days after injury^39^. Semple *et al*. showed (by dendritic lengths, branching, and Sholl intersections) that male mice were more sensitive to CCI induced reductions in prefrontal cortex layer III pyramidal neurons and dentate gyrus granule cells^40^ and these morphological changes were reflected in spatial memory deficits and hyperactivity in similarly treated mice in their study. In another recent blast TBI mouse model, Konan *et al*. observed decreased synapse density within the cortex, which mirror our results indicating a reduction in the dendritic arbor within the cortex^41^. In a previous study using the same blast TBI mouse model, myelinated axonal damage and mitochondrial abnormalities were also observed^42^. These morphological changes were also accompanied by deficits in neurobehavioral outcomes.

### Limitations and strengths

This study provides useful information regarding the morphological changes observed within the cortex and hippocampus following blast TBI during the acute phase. However, it would be useful to characterize temporal changes in response to the injury at longer intervals. Future studies should cover an extended time course and provide increased sample sizes to further strengthen statistical significance. Additionally, the inclusion of detailed morphological analyses, including quantification and categorization of dendritic spines within the regions of interest, would further add to our understanding of post-injury phenomena. Despite these limitations, the findings of this study provide a useful basis for future study of morphological changes following blast TBI.

## Conclusion

These results from our blast TBI model provide an important connection between morphological changes within the brain and associated behavioral and cognitive changes which have been previously observed. Losses in neuronal connectivity can be a major contributor to the symptoms observed after injury^43–45^. In previous studies using our model, we have observed increases in motor dysfunction and anxiety-like behavior, as well as deficits in spatial and recognition memory in blast-injured mice^27,28,46^. We have seen decreased complexity of the dendritic arbor in parietal cortex and hippocampus which suggests a morphological change which could account for the cognitive and memory deficits observed previously, as well as in blast-injured humans^29,47–50^.

## Data Availability

The datasets generated during and/or analyzed during the current study are available from the corresponding author on reasonable request.

## References

[1] Faul, M., Xu, L., Wald, M. M. & Coronado, V. G. Traumatic Brain Injury in the United States: Emergency Department Visits, Hospitalizations and Deaths 2002–2006. (Centers for Disease Control and Prevention, National Center for Injury Prevention and Control, Atlanta, GA, 2010).

[2] Iverson GL (2005). Outcome from mild traumatic brain injury. Curr. Opin. Psychiatry.

[3] Shetty T (2018). Clinical Findings in a Multicenter MRI Study of Mild TBI. Frontiers in neurology.

[4] Isokuortti H (2018). Characterizing the type and location of intracranial abnormalities in mild traumatic brain injury. J. Neurosurg..

[5] Scalea, T. M. In *Neurotrauma: Evidence-based Answers to Common Questions* (eds. Valadka, A. B. & Andrews, B. T.) (Thieme, 2005).

[6] Granacher, R. P. *Traumatic Brain Injury: Methods for Clinical and Forensic Neuropsychiatric Assessment, Second Edition*. (Taylor & Francis Group, 2008).

[7] Defense and Veterans Brain Injury Center. (ed. Department of Defense) 1–5 (Washington, DC, 2018).

[8] Hoge CW (2008). Mild traumatic brain injury in U.S. Soldiers returning from Iraq. N. Engl. J. Med..

[9] Masel BE (2012). Galveston Brain Injury Conference 2010: clinical and experimental aspects of blast injury. J. Neurotrauma.

[10] Elder GA, Christian A (2009). Blast‐related mild traumatic brain injury: mechanisms of injury and impact on clinical care. Mount Sinai Journal of Medicine: A Journal of Translational and Personalized Medicine.

[11] Hoffman SW, DePalma RG, Cifu DX (2017). Veteran’s affairs traumatic brain injury conference: State of the artIntroduction to special edition of brain injury: Guest editors. Brain Inj..

[12] Moore DF (2009). Computational biology - modeling of primary blast effects on the central nervous system. Neuroimage.

[13] Hoffer ME (2010). Blast exposure: vestibular consequences and associated characteristics. Otol. Neurotol..

[14] Shively SB, Perl DP (2017). Viewing the Invisible Wound: Novel Lesions Identified in Postmortem Brains of U.S. Service Members With Military Blast Exposure. Mil. Med..

[15] Robinson ME (2019). Positron emission tomography of tau in Iraq and Afghanistan Veterans with blast neurotrauma. NeuroImage. Clinical.

[16] Ryan LM, Warden DL (2003). Post concussion syndrome. International review of psychiatry (Abingdon, England).

[17] Thompson JM, Scott KC, Dubinsky L (2008). Battlefield brain: unexplained symptoms and blast-related mild traumatic brain injury. Can. Fam. Physician.

[18] Hoffer ME, Donaldson C, Gottshall KR, Balaban C, Balough BJ (2009). Blunt and blast head trauma: different entities. The international tinnitus journal.

[19] Courtney AC, Courtney MW (2009). A thoracic mechanism of mild traumatic brain injury due to blast pressure waves. Med. Hypotheses.

[20] Hoskison MM (2009). Persistent working memory dysfunction following traumatic brain injury: evidence for a time-dependent mechanism. Neuroscience.

[21] Ip EY, Giza CC, Griesbach GS, Hovda DA (2002). Effects of enriched environment and fluid percussion injury on dendritic arborization within the cerebral cortex of the developing rat. J. Neurotrauma.

[22] Casella EM (2014). Traumatic brain injury alters long-term hippocampal neuron morphology in juvenile, but not immature, rats. Childs Nerv. Syst..

[23] Winston CN (2013). Controlled cortical impact results in an extensive loss of dendritic spines that is not mediated by injury-induced amyloid-beta accumulation. J. Neurotrauma.

[24] Gao X, Deng P, Xu ZC, Chen J (2011). Moderate traumatic brain injury causes acute dendritic and synaptic degeneration in the hippocampal dentate gyrus. PLoS ONE.

[25] Gao X, Chen J (2011). Mild traumatic brain injury results in extensive neuronal degeneration in the cerebral cortex. J. Neuropathol. Exp. Neurol..

[26] Campbell JN, Register D, Churn SB (2012). Traumatic brain injury causes an FK506-sensitive loss and an overgrowth of dendritic spines in rat forebrain. J. Neurotrauma.

[27] Rachmany L (2017). Exendin-4 attenuates blast traumatic brain injury induced cognitive impairments, losses of synaptophysin and *in vitro* TBI-induced hippocampal cellular degeneration. Sci Rep.

[28] Rubovitch V (2011). A mouse model of blast-induced mild traumatic brain injury. Exp. Neurol..

[29] Tweedie D (2016). Blast traumatic brain injury-induced cognitive deficits are attenuated by preinjury or postinjury treatment with the glucagon-like peptide-1 receptor agonist, exendin-4. Alzheimer’s & dementia: the journal of the Alzheimer’s Association.

[30] Ratliff WA (2019). Mild blast-related TBI in a mouse model alters amygdalar neurostructure and circuitry. Exp. Neurol..

[31] Diamond DM (2006). Influence of predator stress on the consolidation versus retrieval of long-term spatial memory and hippocampal spinogenesis. Hippocampus.

[32] Shingo AS, Mervis RF, Kanabayashi T, Kito S, Murase T (2015). The dendrites of granule cell layer neurons are the primary injury sites in the “Brain Diabetes” rat. Behav. Brain Res..

[33] Shim SS, Hammonds MD, Mervis RF (2013). Four weeks lithium treatment alters neuronal dendrites in the rat hippocampus. Int. J. Neuropsychopharmacol..

[34] Sholl DA (1953). Dendritic organization in the neurons of the visual and motor cortices of the cat. J. Anat..

[35] McDonald AJ, Mott DD (2017). Functional neuroanatomy of amygdalohippocampal interconnections and their role in learning and memory. J. Neurosci. Res..

[36] Mayer AR, Bellgowan PS, Hanlon FM (2015). Functional magnetic resonance imaging of mild traumatic brain injury. Neurosci. Biobehav. Rev..

[37] Pagulayan, K. F. *et al*. Effect of blast-related mTBI on the working memory system: a resting state fMRI study. *Brain Imaging Behav*., 1–12 (2018). 10.1007/s11682-018-9987-9 PMC11835419 30519997

[38] Ferrante M, Migliore M, Ascoli GA (2013). Functional impact of dendritic branch-point morphology. J. Neurosci..

[39] Thomas TC (2018). Does time heal all wounds? Experimental diffuse traumatic brain injury results in persisting histopathology in the thalamus. Behav. Brain Res..

[40] Semple BD, Dixit S, Shultz SR, Boon WC, O’Brien TJ (2017). Sex-dependent changes in neuronal morphology and psychosocial behaviors after pediatric brain injury. Behav. Brain Res..

[41] Konan LM (2019). Multi-Focal Neuronal Ultrastructural Abnormalities and Synaptic Alterations in Mice after Low-Intensity Blast Exposure. J. Neurotrauma.

[42] Song H (2018). Ultrastructural brain abnormalities and associated behavioral changes in mice after low-intensity blast exposure. Behav. Brain Res..

[43] Rafols JA, Morgan R, Kallakuri S, Kreipke CW (2007). Extent of nerve cell injury in Marmarou’s model compared to other brain trauma models. Neurol. Res..

[44] Scheff S (2005). Synaptogenesis in the hippocampal CA1 field following traumatic brain injury. J. Neurotrauma.

[45] Semchenko V, Bogolepov N, Stepanov S, Maksimishin S, Khizhnyak A (2006). Synaptic plasticity of the neocortex of white rats with diffuse-focal brain injuries. Neurosci. Behav. Physiol..

[46] Tweedie D (2013). Changes in mouse cognition and hippocampal gene expression observed in a mild physical- and blast-traumatic brain injury. Neurobiol. Dis..

[47] Cernak I (2011). The pathobiology of blast injuries and blast-induced neurotrauma as identified using a new experimental model of injury in mice. Neurobiol. Dis..

[48] Haran, F. J., Handy, J. D., Servatius, R. J., Rhea, C. K. & Tsao, J. W. Acute neurocognitive deficits in active duty service members following subconcussive blast exposure. *Applied neuropsychology. Adult*, 1–13 (2019). 10.1080/23279095.2019.1630627 31269805

[49] Ivanov I (2017). Blast Exposure, White Matter Integrity, and Cognitive Function in Iraq and Afghanistan Combat Veterans. Frontiers in neurology.

[50] Pagulayan KF (2018). Retrospective and Prospective Memory Among OEF/OIF/OND Veterans With a Self-Reported History of Blast-Related mTBI. J. Int. Neuropsychol. Soc..

